# Problem gambling severity in a nationally representative sample of the Israeli population: the moderating role of ethnonational affiliation

**DOI:** 10.3389/fpubh.2023.1233301

**Published:** 2023-09-20

**Authors:** Belle Gavriel-Fried, Amit Loewenthal, Noa Vana

**Affiliations:** The Bob Shapell School of Social Work, Tel Aviv University, Tel Aviv, Israel

**Keywords:** problem gambling severity, ethnonational, representative sample, Israel, risk factors

## Abstract

**Introduction:**

Gambling is a public health concern. Problem gambling is related to a range of psycho-social risk factors including ethnonational affiliation. Israel is an insightful case to probe problem gambling, given the socio-economic marginalization of Israeli Arabs, the continued exposure of Israelis to stress and the conservative Israeli gambling market. This study aimed to estimate problem gambling severity in Israeli society, characterize the sociodemographic, environmental, mental health, and gambling-related risk factors associated with problem gambling severity, and examine the role of ethnonational affiliation (Israeli Jewish/Arab) as a moderating factor in the associations between these risk factors and problem gambling severity.

**Methods:**

A cross-sectional study based on a representative sample of 3,244 Israelis was conducted in 2022, of whom 1,626 had gambled in the previous year. Measurements included Problem Gambling Severity Index, Gambling Behaviors, Perceived Neighborhood Cohesion, Stress, the Patient Health Questionnaire-4, and sociodemographic variables.

**Results:**

Low-risk, moderate-risk and problem gambling were reported by 16.7% of the total sample. The main risk factors for problem gambling were being an Israeli Arab, traditional, residing in a low socio-economic cluster, reporting anxiety symptoms, and higher gambling behaviors, specifically in legal/illegal online gambling. Ethnonational affiliation (Israeli Jewish/Arab) moderated the associations between gambling, illegal online gambling, and problem gambling severity. Higher neighborhood cohesion emerged as a risk factor for problem gambling solely for Israeli Arabs.

**Discussion:**

As an excluded minority, Israeli Arabs may turn to gambling as a method to access the core republican community, thus, exposing themselves to a higher risk of developing problem gambling.

## 1. Introduction

Gambling constitutes a challenge to public health due to its adverse consequences to society and individuals who engage in frequent, uncontrolled gambling (1). Problem gambling (PG) is defined as the accumulation of negative consequences in a range of facets of life as a result of gambling in a given period of time (2). The prevalence rates of PG worldwide have been estimated at 0.12% to 5.8% (1). Classically, PG ranges on a problem gambling severity (PGS) continuum from low-risk gamblers to moderate-risk to problem gamblers (3). Studies show that individuals with a higher level of PGS report higher harm levels than individuals with lower PGS (4).

PG is associated with ethnocultural (5), sociodemographic (6), mental-health risk factors (7), and type of gambling (2). Studies have found higher rates of gambling and PGS in certain ethnic minorities (e.g., Afro-Americans, Hispanics, Asians, and Arabs) than in majority groups (e.g., people with European backgrounds) who gamble (5, 8–11). This may be attributed to socio-economic issues such as language barriers and social isolation (12), as well as specific ethnocultural and religious values and beliefs (13) that may increase stress and depression during the acculturation processes of ethnocultural minorities (14), as well as residence in disadvantaged neighborhoods (5). Online gambling and gambling frequency are also considered greater risk factors for minorities (8).

The literature has consistently identified associations between PG and sociodemographic variables including male gender, young age (6), fewer years of education (9), and between higher levels of PGS and being secular (15). Residing in socioeconomically disadvantaged areas with high rates of unemployment and poor households is also associated with an increased risk of PG (5, 16). PG is also negatively associated with self-perceived characteristics of place of residence, feelings of personal safety (17), trust (18), and sense of community (10). These characteristics are all embedded in individuals’ self-perceived neighborhood social cohesion (19), which to the best of our knowledge has never been explored in relation to PG.

Anxiety and depression are commonly associated with PG (20), as well as exposure to stress (7). Stress and anxiety may trigger gambling, but gambling can also become a stressor (21). Problem gamblers may gamble to alleviate depression and anxiety (20). A recent meta-analysis of risk factors for PG found that continuous modes of gambling, such as internet gambling, led to a higher odds ratio for developing PG (2). Individuals involved in online-only games and mixed-mode gambling (i.e., land-based and online gambling) had higher levels of PGS than individuals engaged in land-based only gambling (22). Self-reported illegal gambling was also associated with higher levels of PGS in a non-representative sample of Israelis (23).

This study examined a representative sample of residents of the State of Israel. It was designed to characterize and identify the sociodemographic, environmental, psychopathological, and gambling-related risk factors associated with PGS in Israeli society and explore the role of ethnonational affiliation (Israeli Jewish/Arab) as a moderating factor in the relationship between environmental, psychopathological, and gambling-related variables and PGS. PGS has been documented in representative samples in Israel by the Israel Authority for the Prevention of Violence, Alcohol and Drug Abuse which includes the PGSI in its survey that mainly assesses the use of psychoactive substances (24). Surveys have also been conducted every year since 2014 for the Israeli lottery by a private company, whose main findings are summarized on the Israeli lottery website (25). However, the findings on PGS in these surveys have not been published in peer reviewed journals, and PGS has not been examined in terms of the specific characteristics of Israeli society.

Israel constitutes a valuable setting to research the relationship between sociocultural contexts and PGS. First, Israel is populated by two groups with different ethnonational affiliations: the majority group of Israeli Jews, and the minority group of Israeli Arabs, the umbrella term for Christian, Druze, and mostly Muslim Palestinian Arabs who are Israeli citizens, and make up 20% of Israel’s population (26). Israel was founded to create a national home for its Jewish majority which grants the Jewish population with collective and individual privileges that do not extend to Israel’s ethnonational minority group; i.e., Israeli Arabs who are geographically, socially, economically, and institutionally marginalized (26, 27). There are only a few empirical studies on Arabs and PG (28, 29). A handful have examined PG in Arab countries such as Lebanon and Morocco (30, 31), while several others have investigated PG among Arab minorities in Western countries such as Canada (11). Several studies have investigated PG in Arab minorities in Western countries such as Canada (11). Hence, the current study provides an opportunity to shed light on this field, and specifically on Arabs as an ethnonational and marginalized minority group. Second, the Israeli population as a whole is also exposed to the longstanding Israeli-Palestinian conflict (32) which generates continuous stressors and trauma (33). Their association with stress, anxiety, and depression is well-documented in the Israeli context (32) and elsewhere (34). Finally, the gambling market in Israel is considered conservative, due in part to the Jewish religious sector’s aversion to gambling, as made explicit by Israel’s religious political parties (35) and most online gambling is illegal (36).

Based on the literature review, five hypotheses were formulated:

*H1:* Sociodemographic variables: being a man, Israeli Arab, young, and secular, with a low level of education, and unemployed will be associated with higher levels of PGS.

*H2:* Environmental variables: respondents reporting low perceived neighborhood cohesion and residing in a low socioeconomic cluster will present higher levels of PGS.

*H3:* Psychopathological variables: stress, depression, and anxiety will be associated with higher levels of PGS.

*H4:* Gambling behaviors: a higher frequency of involvement in gambling behaviors and engagement in legal and illegal online gambling will be associated with higher levels of PGS.

*H5:* Ethnonational affiliation (Israeli Jewish/Arab) will moderate the associations between environmental, psychopathological, and gambling-related characteristics and higher levels of PGS. Specifically, Israeli Arabs living in lower socioeconomic clusters, who report lower perceived neighborhood cohesion and more depression, anxiety, and stress, who are more involved in gambling behaviors, including legal and illegal online gambling would be more likely to present higher levels of PGS than Israeli Jews with the same environmental, psychopathological and gambling-related characteristics.

## 2. Method

### 2.1. Sample and procedure

This study is part of a larger research project on gambling behaviors in Israeli adults employing a web-based representative random sample of the Israeli population. The BI and the Lucille Cohen Institute for Public Opinion Research at Tel Aviv University constructed the panel according to methodological standards for web-based studies defined by the National Opinion Research Center at the University of Chicago and the PEW Research Institute for studies on Israeli society (37). The panel included a stratum for the Jewish population and a stratum for the Arab population reflecting Israel’s ethnonational stratification. The Israeli Jews were randomly selected from the population registry database. Their phone numbers were then located. By contrast, it was virtually impossible to pinpoint phone numbers of specific Israeli Arabs. Hence, households of Israeli Arabs were randomly selected out of the household database. The gender and age distribution of the Israeli Jewish individuals in the panel was similar to that of the Jewish population in Israel (except for 70+ year-olds, who were underrepresented). College graduates were overrepresented, whereas the Ultra-Orthodox were underrepresented. No similar administrative data on the gender and age distribution was available for the Israeli Arabs (37).

For purposes of the current study, an invitation and six reminders with a link to the survey (in Hebrew for Israeli Jews and in Arabic for Israeli Arabs) were sent to all panel members between July and September 2022 which comprised 10,499 Israeli Jews and 1,302 Israeli Arabs. The response rate was 30.9% for the Israeli Jews, and 11.7% for the Israeli Arabs, for a total of 3,080 Israeli Jews and 164 Israeli Arabs. The proportions of age and gender in the sample were consistent with the general distribution of these variables in the Israeli population. After the sampling process, the two groups were merged and post-weighted according to their proportions in the general population and levels of education. The current study included 1,626 Israeli citizens (790 women; 57 Israeli Arabs), aged 18 and older, who had gambled at least once in the previous year (Table 1).

**Table 1:** Descriptive statistics of a nationally representative sample of Israelis who engaged in gambling, 2022 (*N* = 1,626).

Variable	Full sample		Jews		Arabs				
**Demographic variables**	**%**		**%**		**%**		**χ**^**2**^	***p*-value**	**Cramér’s V**
*Gender*							38.031	< 0.001	0.154
Woman	44.8%		48.1%		26.8%				
Man	55.2%		51.9%		73.2%				
*Ethnonational affiliation*							–		–
Israel Jewish	84.7%		–		–				
Israeli Arab	15.3%		–		–				
*Self-perceived religiosity*							91.386	< 0.001	0.239
Secular	40.7%		44.1%		21.6%				
Traditional	42.4%		37.4%		70.2%				
Religious or very religious	16.9%		18.5%		8.2%				
*Education*							33.115	< 0.001	0.144
Non-academic	67.8%		65.0%		83.7%				
Academic	32.2%		35.0%		16.3%				
*Labor market status*							89.276	< 0.001	0.236
Employed	73.6%		75.5%		62.9%				
Unemployed	6.0%		3.6%		19.2%				
Not in the labor force	20.4%		20.9%		18.0%				
*Age group*							15.316	0.002	0.98
18–29	21.6%		22.9%		14.6%				
30–39	23.5%		22.1%		31.3%				
40–64	43.9%		43.7%		45.1%				
65+	11.0%		11.4%		8.9%				
**Environmental variables**	**Mean**	**SD**	**Mean**	**SD**	**Mean**	**SD**	***t***	***p*-value**	**Cohen’s D**
Locality’s socioeconomic cluster (N = 1,610)	5.86	2.24	6.24	2.07	3.63	1.94	18.403	< 0.001	1.272
Neighborhood cohesion	16.72	3.50	16.84	3.47	16.06	3.63	3.110	0.002	0.223
**Psychological and psychopathological variables**	**%**		**%**		**%**		**χ**^**2**^	***p*-value**	**Cramér’s V**
Depression	29.7%		26.1%		49.8%		56.040	< 0.001	0.187
Anxiety	21.4%		17.2%		44.9%		94.543	< 0.001	0.243
	**Mean**	**SD**	**Mean**	**SD**	**Mean**	**SD**	***t***	***p*-value**	**Cohen’s D**
Stress	4.36	2.92	4.12	2.69	5.70	3.68	−6.450	< 0.001	−0.554
**Gambling variables**	**%**		**%**		**%**		**χ**^**2**^	***p*-value**	**Cramér’s V**
*Problem gambling severity*							109.052	< 0.001	0.261
Non-problem gamblers	66.2%		71.2%		38.2%				
Low-risk gamblers	21.0%		18.4%		35.4%				
Moderate-risk gamblers	10.1%		8.6%		19.1%				
Problem gamblers	2.7%		1.8%		7.3%				
Legal online gambling	12.4%		13.2%		8.2%		4.853	0.028	0.055
Illegal online gambling	8.7%		6.8%		19.1%		39.893	< 0.001	0.158
	**Mean**	**SD**	**Mean**	**SD**	**Mean**	**SD**	***t***	***p*-value**	**Cohen’s D**
Gambling behaviors	1.27	0.22	1.26	0.22	1.31	0.20	−3.674	< 0.001	−0.234

The data were weighted by level of education, and ethnonational affiliation (Israeli Jewish/Arab).

### 2.2. Measures

#### 2.2.1. Problem gambling severity index

This nine-item scale measures the extent of PGS in general population surveys (3). Its 4-point scale ranges from 0 (never) to 3 (almost always). Participants are classified into four severity categories: 0 = ‘Non-problem gamblers’; 1–2 = ‘Low-risk gamblers’; 3–7 = ‘Moderate-risk gamblers’; and 8+ = ‘Problem gamblers’. Note that previous epidemiological studies have merged ‘moderate-risk gamblers’ and ‘problem gamblers’ into one category (38–41). The Cronbach’s alpha for this scale was 0.832 for the Israeli Jewish respondents and 0.696 for the Israeli Arab respondents.

#### 2.2.2. Gambling behaviors

A scale was developed for this study covering 11 common types of gambling in Israel and an additional item labelled “other” to capture unspecified types of gambling. The respondents were asked to indicate how often they gambled for each type on a five-point Likert scale (1 = “Not at all” 5 = once a week or more’).

The participants’ overall gambling involvement score was calculated as the mean of all 12 gambling types. We also calculated legal and illegal online gambling scores as binary indicators representing the respondents’ engagement in these gambling types over the past year. Legal gambling was defined as state-regulated gambling, gambling when abroad, and gambling on the stock market. Illegal gambling covered all non-regulated forms of gambling, land-based gambling venues and gambling online (except gambling on the stock market). Supplementary Table S1 details the types of gambling, in terms of regulator, modes, and legality.

#### 2.2.3. Perceived neighborhood cohesion

A five-item scale that measures self-perceived neighborhood social cohesion (19). The respondents were asked to rank cohesion on a five-point Likert scale (1-strongly disagree; 5-strongly agree). The total sum was calculated. The Cronbach’s alpha was 0.732 for the Jewish Israeli respondents and 0.667 for the Arab Israeli respondents.

#### 2.2.4. Subjective stress

A single item assessing subjective stress. Respondents rated how much stress they had felt in the past month on a 0–10 scale, where 10 indicates extreme stress.

#### 2.2.5. Patient health Questionnaire-4

This ultra-brief scale measures anxiety and depression. Two items measure levels of generalized anxiety, GAD-2, and two measure depression, PHQ-2 (42). Responses are rated on a four-point scale from not at all (= 0) to nearly every day (= 3). The Cronbach’s alpha for GAD-2 was 0.851 for the Israeli Jewish sample and 0.943 for the Israeli Arab sample. For the PHQ-2, the Cronbach’s alpha was 0.544 and 0.708, respectively.

#### 2.2.6. Socioeconomic cluster

The SES of the respondents’ place of residence was classified according to the residence locality measure developed by the Israel Central Bureau of Statistics. The data ranged from (1) the lowest cluster, to (10) the highest cluster (43).

#### 2.2.7. Demographic variables

The sociodemographic variables included gender, ethnonational affiliation, age group, self-perceived religiosity, education, and labor market status.

### 2.3. Data analysis

Frequencies of PGSI categories were calculated for the whole sample (*n* = 3,244). All other analyses were conducted on those who gambled in the past year (*n* = 1,626). In all the analyses, the data were weighted by level of ethnonational affiliation (Israeli Jewish/Arab) and education. Differences between Israeli Jews and Israeli Arabs were assessed with a Pearson Chi-Square test with a Bonferroni correction for binary and categorical variables and a one-way ANOVA with a post-hoc Tukey test for continuous variables. The same analyses were also conducted to compare classification into categories of PGS. Due to the small number of individuals classified as problem gamblers (*n* = 29), the problem gambler category was merged with the moderate-risk gambler category (for a total of *n* = 164).

A multinomial logistic regression was conducted with PGSI (three categories) with sociodemographic variables, socioeconomic cluster, neighborhood cohesion, depression, anxiety, stress, gambling behaviors score, and legal and illegal gambling indicators as predictors. Note that PGSI is an ordinal variable, which usually implies an ordinal logistic regression, but since it failed to meet assumptions for proportional odds, a multinomial logistic regression was used, similar to previous studies that have administered the PGSI (41, 44–46). The independent ordinal variables (socioeconomic cluster, neighborhood cohesion, stress, and gambling behaviors score) were standardized prior to being included in the regression analysis. All other variables were binary or categorical. The reference category for the multinomial regression was non-problem gamblers. To test for moderation, we ran a second regression with the addition of the interactions between ethnonational affiliation and a subset of variables that can be considered as moderated by ethnic minority status according to the literature: environmental variables (5), psychopathological variables (14) and gambling-related variables (8), with the exception of online legal gambling. The small proportion of Israeli Arabs who stated that they engaged in legal online gambling resulted in large CIs for the “Legal online gambling × Arab” interaction. It was therefore excluded from the model. This did not significantly affect the results.

## 3. Results

### 3.1. Descriptive statistics

Of the total sample (*N* = 3,244), 49.3% had gambled in the previous year, of whom 32.6% were classified as non-problem gamblers, 10.4% as low-risk gamblers, and 6.3% as moderate-risk and problem gamblers. Among those who had gambled in the past year, non-problem gamblers comprised 66.2%; 21.0% were low-risk gamblers, 12.8% were moderate-risk and problem gamblers. Individuals who engaged in legal online gambling comprised 12.4% of the sample; 8.6% engaged in illegal online gambling. Roughly 29.7% reported depressive symptoms, and 21.4% reported anxiety symptoms.

The findings also showed statistically significant differences between Israeli Jews and Israeli Arabs (Table 1). Israeli Arabs who engaged in gambling were more likely to be men, traditional, less educated, younger, and unemployed. They also resided in localities with a significantly lower socioeconomic cluster and reported lower perceived neighborhood cohesion. The analyses indicated that 49.8% of the Israeli Arabs who engaged in gambling also reported depressive symptoms, compared to 26.1% of the Israeli Jews who engaged in gambling. The data indicated that 44.9% reported anxiety symptoms, compared to 17.2% of the Israeli Jews. Israeli Arabs reported significantly more stress. Israeli Jews and Israeli Arabs differed in terms of gambling behaviors. Israeli Arabs had higher gambling behaviors scores and engaged more in illegal online gambling, while Israeli Jews turned more to legal online gambling.

### 3.2. Differences between PGS categories

Statistically significant differences were found between all three PGS categories in relation to labor market status and gambling-related variables (Table 2). Problem and moderate-risk gamblers tended to be unemployed, followed by low-risk and non-problem gamblers. Problem and moderate-risk gamblers had a significantly larger share of 18–29 years olds than the other categories. Problem and moderate-risk gamblers were also more likely to be involved in online legal and illegal gambling and had the highest gambling behaviors score, followed by low-risk and non-problem gamblers.

**Table 2:** Differences across PGSI groups of Israelis who engaged in gambling, 2022 (*N* = 1,626).

PGSI	Non-problem gamblers		Low-risk gamblers		Problem and moderate-risk gamblers				
**Demographic variables**	**%**		**%**		**%**		**χ**^**2**^	***p*-value**	**Cramér’s V**
*Gender*							14.128	< 0.001	0.094
Woman	48.1% _a_		39.9% _b_		36.1% _b_				
Man	51.9% _a_		60.1% _b_		63.9% _b_				
*Ethnonational affiliation*							105.145	< 0.001	0.256
Israel Jewish	91.1% _a_		74.1% _b_		68.3% _b_				
Israeli Arab	8.9% _a_		25.9% _b_		31.7% _b_				
*Self-perceived religiosity*							67.954	< 0.001	0.146
Secular	45.9% _a_		33.0% _b_		26.2% _b_				
Traditional	35.4% _a_		52.4% _b_		62.1% _b_				
Religious or very religious	18.7% _a_		14.6% _a, b_		11.7% _b_				
*Education*							25.979	< 0.001	0.127
Non-academic	63.7% _a_		74.1% _b_		79.0% _b_				
Academic	36.3% _a_		25.9% _b_		21.0% _b_				
*Labor market status*							65.352	< 0.001	0.143
Employed	77.0% _a_		66.6% _b_		67.8% _b_				
Unemployed	3.3% _a_		7.8% _b_		16.6% _c_				
Not in the labor force	19.7% _a, b_		25.7% _b_		15.6% _a_				
*Age group*							25.819	< 0.001	0.90
18–29	20.7% _a_		19.0% _a_		30.2% _b_				
30–39	22.4% _a_		26.8% _a_		23.9% _a_				
40–64	47.1% _a_		38.7% _b_		36.1% _b_				
65+	9.8% _a_		15.5% _b_		9.8% _a, b_				
**Environmental variables**	**Mean**	**SD**	**Mean**	**SD**	**Mean**	**SD**	***F***	***p*-value**	**η**^2^
Locality’s socioeconomic cluster (N = 1,610)	6.16 _a_	2.14	5.34 _b_	2.41	5.25 _b_	2.20	26.421	< 0.001	0.033
Neighborhood cohesion	16.86 _a_	3.51	16.69 _a, b_	3.46	16.05 _b_	3.51	4.620	0.010	0.006
**Psychological and psychopathological variables**	**%**		**%**		**%**		**χ**^**2**^	***p*-value**	**Cramér’s V**
Depression	26.1% _a_		37.2% _b_		35.9% _b_		19.336	< 0.001	0.110
Anxiety	17.9% _a_		25.0% _b_		33.7% _b_		28.397	< 0.001	0.133
	**Mean**	**SD**	**Mean**	**SD**	**Mean**	**SD**	***F***	***p*-value**	**η**^2^
Stress	4.14 _a_	2.74	4.67 _b_	2.98	4.96 _b_	3.546	9.253	< 0.001	0.011
**Gambling variables**	**%**		**%**		**%**		**χ**^**2**^	***p*-value**	**Cramér’s V**
Legal online gambling	8.7% _a_		15.8% _b_		26.3% _c_		53.499	< 0.001	0.183
Illegal online gambling	3.7% _a_		7.1% _b_		37.1% _c_		242.886	< 0.001	0.389
	**Mean**	**SD**	**Mean**	**SD**	**Mean**	**SD**	***F***	***p*-value**	**η**^2^
Gambling behaviors	1.22 _a_	0.16	1.35 _b_	0.23	1.40 _c_	0.34	101.857	< 0.001	0.113

The data were weighted by level of education, and ethnonational affiliation (Israeli Jewish/Arab). Each subscript denotes a subset of PGS categories whose column proportions did not differ significantly from each other at the 0.05 level. For example, the subscripts a, b and c for the variable “Legal online gambling” indicate that this variable differs significantly across PGS categories.

Problem and moderate-risk gamblers differed significantly from non-problem gamblers on most demographic, environmental, and psychopathological variables but not from low-risk gamblers. Specifically, problem and moderate-risk gamblers were more likely to be men, traditional, and less likely to be secular, employed, and have an academic degree than non-problem gamblers. Problem and moderate-risk gamblers were more likely to reside in a lower socioeconomic cluster and a neighborhood with lower levels of perceived neighborhood cohesion than non-problem gamblers. Problem and moderate-risk gamblers reported more stress and were more likely to note depression and anxiety symptoms than non-problem gamblers. Problem, moderate-risk, and low-risk gamblers were all more likely to be Israeli Arabs than non-problem gamblers.

### 3.3. Multinomial regression results

The results of the first model appear in Table 3. All the results reported in this subsection and the next took non-problem gamblers as the reference group. Hence the results do not compare low-risk gamblers to problem and moderate gamblers directly. In the first model (Table 3), Israeli Arabs were found to have significantly higher odds of being categorized as low-risk (OR = 2.84; 95% CI = 1.85–4.37), and problem and moderate-risk gamblers (OR = 3.00; 95% CI = 1.75–5.15), compared to Israeli Jews. Being traditional was associated with higher odds of being categorized as a low-risk gambler (OR = 1.44; 95% CI = 1.05–1.98) and being categorized as a problem and moderate-risk gambler (OR = 1.91; 95% CI = 1.24–2.92), compared to being secular. Respondents residing in localities from higher socioeconomic clusters had lower odds of being categorized as low-risk gamblers (OR = 0.78; 95% CI = 0.67–0.92).

**Table 3:** Risk factors for PGS among Israelis who engaged in gambling, 2022 (*N* = 1,610).

Reference category: non-problem gamblers	Low-risk gamblers	Problem and moderate-risk gamblers
	Odds ratio (95% CI)	Odds ratio (95% CI)
Constant	0.22 (0.14–0.34)	0.09 (0.05–0.16)
**Sociodemographic variables**		
Gender (man vs. woman)	0.97 (0.72–1.30)	1.40 (0.95–2.08)
Ethnonational affiliation (Israeli Arab/Jewish)	2.84 (1.85–4.37)	3.00 (1.75–5.15)
*Self-perceived religiosity (reference category: secular)*		
Traditional	1.44 (1.05–1.98)	1.91 (1.24–2.92)
Religious or very religious	0.82 (0.53–1.28)	0.99 (0.54–1.83)
Education (academic vs. non-academic)	0.83 (0.61–1.13)	0.75 (0.49–1.14)
*Labor market status (reference category: employed)*		
Unemployed	1.16 (0.60–2.22)	1.84 (0.90–3.76)
Not in the labor force	1.22 (0.84–1.76)	0.62 (0.36–1.06)
*Age group (reference category: 18–29)*		
30–39	0.96 (0.63–1.46)	0.53 (0.31–0.90)
40–64	0.86 (0.59–1.26)	0.59 (0.37–0.92)
65+	1.48 (0.88–2.47)	1.29 (0.65–2.56)
**Environmental variables**		
Locality’s socioeconomic cluster	0.78 (0.67–0.92)	0.86 (0.69–1.07)
Neighborhood cohesion scale	1.08 (0.94–1.25)	0.96 (0.80–1.17)
**Psychopathological variables**		
Depression	1.18 (0.84–1.65)	0.81 (0.50–1.30)
Anxiety	1.20 (0.80–1.81)	1.74 (1.03–2.95)
Self-reported stress level	1.08 (0.92–1.28)	1.10 (0.88–1.37)
**Gambling-related variables**		
Legal online gambling	1.73 (1.14–2.63)	2.67 (1.65–4.32)
Illegal online gambling	1.11 (0.61–2.02)	7.51 (4.46–12.62)
Gambling behavior	1.92 (1.65–2.25)	1.75 (1.46–2.10)

In this multinomial logit regression, “non-problem gamblers” was the reference category. Multicollinearity was adequate: all VIF ≤ 1.65. The data were weighted by level of education, and ethnonational affiliation (Israeli Jewish/Arab).

Respondents reporting anxiety symptoms had higher odds of being categorized as problem and moderate-risk gamblers (OR = 1.74; 95% CI = 1.03–2.95). Respondents who engaged in legal online gambling had higher odds of being categorized as low-risk gamblers (OR = 1.73; 95% CI = 1.14–2.63) and being categorized as problem and moderate-risk gamblers (OR = 2.67; 95% CI = 1.65–4.32). By contrast, respondents who engaged in illegal online gambling had higher odds of being categorized as problem and moderate-risk gamblers (OR = 7.51; 95% CI = 4.46–12.62), but not as low-risk gamblers (OR = 1.11; 95% CI = 0.61–2.02). Respondents with a higher gambling behaviors score had higher odds of being categorized as low-risk gamblers (OR = 1.92; 95% CI = 1.65–2.25) and problem and moderate-risk gamblers (OR = 1.75; 95% CI = 1.46–2.10).

### 3.4. Moderation test results

The moderating role of ethnonational affiliation was partially confirmed (Table 4). There was a stronger association between perceived neighborhood cohesion in Israeli Arabs and being categorized as a low-risk gambler (OR = 5.04; 95% CI = 2.51–10.14) and being characterized as a problem and moderate-risk gambler (OR = 2.53; 95% CI = 1.06–6.05), but not among Israeli Jews (OR = 0.92; 95% CI = 0.78–1.07, and OR = 0.89; 95% CI = 0.72–1.10, respectively). There was also a stronger association for Israeli Arabs between being categorized as low-risk gamblers, and the gambling behaviors score (OR = 4.45; 95% CI = 1.93–10.29) than Israeli Jews (OR = 1.77; 95% CI = 1.50–2.09). There was also a stronger association for Israeli Arabs between illegal online gambling and being categorized as moderate-risk or problem gamblers (OR = 231.01; 95% CI = 7.14–7469.17). Note that the Israeli Arab group was small, leading to high uncertainty in the estimation of this association, resulting in wide CIs, compared to Israeli Jews (OR = 3.94; 95% CI = 2.15–7.22). In contrast, Israeli Arabs reporting depressive symptoms were less likely to be categorized as moderate-risk or problem gamblers (OR = 0.02; 95% CI = 0.00–0.17) than Israeli Jews (OR = 1.39; 95% CI = 0.86–2.25).

**Table 4:** Risk factors for PGS moderated by ethnonational affiliation (Arab) among Israelis who engaged in gambling, 2022 (*N* = 1,610).

Reference category: non-problem gamblers	Low-risk gamblers	Problem and moderate-risk gamblers
	Odds ratio (95% CI)	Odds ratio (95% CI)
Locality’s socioeconomic cluster × Arab	0.55 (0.26–1.17)	0.65 (0.28–1.54)
Neighborhood cohesion scale × Arab	5.04 (2.51–10.14)	2.53 (1.06–6.05)
Depression × Arab	1.67 (0.32–8.81)	0.02 (0.00–0.17)
Anxiety × Arab	0.24 (0.05–1.18)	1.62 (0.27–9.56)
Self-reported stress × Arab	0.55 (0.30–1.02)	0.72 (0.36–1.43)
Illegal online gambling × Arab	16.82 (0.51–554.13)	231.01 (7.14–7469.17)
Gambling behaviors × Arab	4.45 (1.93–10.29)	2.47 (0.93–6.58)

In this multinomial logit regression, “non-problem gamblers” was the reference category. Other variables are presented in Supplementary Table S2 in the supplementary material. The data were weighted by level of education, and ethnonational affiliation (Israeli Jewish/Arab).

## 4. Discussion

This study is the first to examine PGS categories in relation to the specific characteristics of Israeli society based on a representative sample of Israeli residents. The findings showed higher rates of gambling behavior scores and PGS among Israeli Arabs than Israeli Jews. The data indicated that being an Israeli Arab was a predictor of PGS and a moderator in the association between environmental, psychopathological, and gambling behaviors variables and PGS. Israeli Arabs with higher gambling behaviors scores and higher levels of neighborhood cohesion fell primarily into the low-risk, and problem and moderate risk gambling categories. Involvement in illegal online gambling increased the probability for Israeli Arabs to be classified into the problem and moderate risk gambling categories. Surprisingly, Israeli Arabs reporting higher levels of depression were less likely to be classified in higher PGS categories.

These findings are consistent with previous studies showing that membership in an ethnic minority constitutes a risk factor for PG (8). These findings may be explained by the characteristics of the Israeli Arab minority. Although by law, Israeli Arabs have individual human and civil rights they are denied participation in the core republican community (47). Their willingness and ability to actively exercise citizenship; i.e., formulate community goals and share its resources equally has made them second-class citizens (48). Hence, as an excluded minority, Israeli Arabs strive to use every possible resource to raise their socioeconomic status and achieve some form of equality with the Jewish majority (49). Thus, greater involvement in gambling behaviors may be a way to access the core republican community through wealth acquired by gambling, since everyone is theoretically equal in games of luck. Israeli Arabs’ decision to gamble illegally online might be a manifestation of their active and vocal opposition to the Jewish state since gambling illegally online reflects their unwillingness to share their potential profits from gambling with the state through taxes. Numerous studies have addressed feelings of shame related to gambling (50, 51). Given Islam’s explicit prohibition against gambling (52), it is not surprising that the few studies exploring Arabs and Muslims have addressed shame in relation to PG (13, 28, 53). Hence, online gambling may be a way to conceal their involvement in gambling.

The Israeli Arabs are divided in their relation to the Israeli State. Some strive to “break free,” change their lives, and acquire equal rights and participation in the state’s resources. However, others have given up, boycotting Israeli elections, refusing to participate in the public sphere, and segregating themselves within their communities (54). While the literature usually depicts depression as a risk factor for PG (20), the findings here suggest differently. Specifically, in the Israeli context, the association between depression and higher PGS categories appeared to be moderated by ethnonational affiliation. Israeli Arabs reporting higher levels of depression, due to their despair from their ongoing oppression (54), may thus be less likely to engage in gambling. They may not believe there is any possible horizon for change in their lives so that gambling does not hold any appeal for them.

Israeli Arabs tend to reside near their extended families and participate in their clan’s activities (54). Recently, higher crime rates among Israeli Arabs have been associated with organized crime led by dominant clans (27). Israeli Arabs could possibly thus feel higher levels of neighborhood cohesion and simultaneously be more prone to becoming risk gamblers as a result of their clan’s possible involvement in non-normative behaviors including illegal gambling.

In terms of Israeli society as a whole, the findings indicate that anxiety, legal and illegal online gambling, and greater involvement in gambling were associated with more severe categories of PGS, as found in previous studies on well-known risk factors for PG (2, 8, 20, 23). The results here also showed that residing in a low socioeconomic cluster was associated with higher categories of PGS. We predicted that a socioeconomic disadvantage might be a risk factor for PG, similar to previous findings (5). Contrary to other studies (15), respondents who defined themselves as religiously traditional were more likely to fall into higher PGS categories than secular respondents. This may stem from characteristics related to having self-perceived traditional views in the Israeli context.

There was no significant association between stress and PGS. Israelis are exposed to continuous stressors, but this was not reflected in the respondents’ PGS categories. Since gambling is a less accepted activity in Israeli society than in other developed countries (55) it might not be considered a legitimate way to mitigate stress.

This study presented the prevalence of PGS in Israeli society based on a nationally representative sample. Israel has a relatively conservative gambling policy (55). Surprisingly, the findings revealed that the prevalence of PG in Israel falls within the range of PG reported in other nationally representative samples (1). The country’s conservative policy which is meant to protect the population from the risks inherent to gambling may in fact mask public awareness of these risks, and by doing so expose them inadvertently to gambling problems.

### 4.1. Public health implications

Future studies should include and proportionally represent minority groups in national representative samples. Effective policies should promote and raise awareness of PG and should instigate restrictions on online gambling. Culturally sensitive prevention and treatment programs should be developed for minorities, and specifically for Muslim and Arab groups, as well as targeted campaigns to increase their awareness of PG and reduce feelings of shame related to PG. In the Israeli context, policymakers should provide legitimate and accessible modes of public participation and set state policies for Israeli Arabs.

### 4.2. Research limitations

This is a self-report cross-sectional study. For the Israeli Arab panel, the response rate was low (11.7%), resulting in fewer observations of Israeli Arabs than their representative share in the population. This low response rate is consistent with population surveys that have included ethnonational minorities in other countries (56), and studies that have used the current panel (57). Future studies should use different methods to reach more Israeli Arab respondents and capture broader perspectives on Arab society in Israel. Despite these limitations, this study sheds light on gambling in Israel, enriches the public health literature on Arabs and problem gambling, and highlights the importance of including ethnic minority groups in national representative samples.

## Data availability statement

The original contributions presented in the study are included in the article/Supplementary material, further inquiries can be directed to the corresponding author.

## Ethics statement

This study was approved by the institutional review board of Tel Aviv University (approval number 0002764-3). Participants provided their written informed consent to participate in the study.

## Author contributions

BG-F led the research from the ground up, formulated the research aims, and conceptualized the research model. She was responsible for the study design and data collection and was involved in writing the manuscript. AL conducted the data analysis and was involved in writing the manuscript. NV was involved in writing the discussion. All authors have approved the final version.

## Funding

The current study was funded by a grant from the Committee for Independent Studies of the National Lottery of Israel awarded to BG-F. These grants did not influence the current study.

## Conflict of interest

BG-F received grants from the Israel National Insurance Institute and the Committee for Independent Studies of the National Lottery of Israel. A grant for independent studies from the National lottery of Israel funded AL. A grant for independent studies from the National Lottery of Israel funded NV. However, these funding sources did not influence this study, and the funding institutions played no role at any stage.

## Publisher’s note

All claims expressed in this article are solely those of the authors and do not necessarily represent those of their affiliated organizations, or those of the publisher, the editors and the reviewers. Any product that may be evaluated in this article, or claim that may be made by its manufacturer, is not guaranteed or endorsed by the publisher.

## References

[1] Calado F Griffiths MD. Problem gambling worldwide: an update and systematic review of empirical research (2000–2015). J Behav Addict. (2016) 5:592–613. doi: 10.1556/2006.5.2016.073, PMID: 27784180 PMC5370365

[2] Allami Y Hodgins DC Young M Brunelle N Currie S Dufour M. A meta-analysis of problem gambling risk factors in the general adult population. Addiction. (2021) 116:2968–77. doi: 10.1111/add.15449, PMID: 33620735 PMC8518930

[3] Ferris J Wynne H. The Canadian problem gambling index. Ottawa, ON: Canadian Center on Substance Abuse (2001).

[4] Delfabbro P Georgiou N King DL. Measuring gambling harm: the influence of response scaling on estimates and the distribution of harm across PGSI categories. J Gambl Stud. (2021) 37:583–98. doi: 10.1007/s10899-020-09954-1, PMID: 32424665

[5] Barnes GM Welte JW Tidwell MCO. Gambling involvement among native Americans, blacks, and whites in the United States. Am J Addict. (2017) 26:713–21. doi: 10.1111/ajad.12601, PMID: 28782902 PMC5610650

[6] Volberg RA Williams RJ Stanek EJ Houpt A Zorn M Rodriguez-Monguio R. Gambling and problem gambling in Massachusetts: Results of a baseline population survey. Amherst, MA: School of Public Health and Health Sciences, University of Massachusetts Amherst (2017).

[7] Ronzitti S Kraus SW Hoff RA Potenza MN. Stress moderates the relationships between problem-gambling severity and specific psychopathologies. Psychiatry Res. (2018) 259:254–61. doi: 10.1016/j.psychres.2017.10.028, PMID: 29091825 PMC5742031

[8] Caler KR Vargas Garcia JR Nower L. Problem gambling among ethnic minorities: results from an epidemiological study. Asian J. Gambl. Issues Public Health. (2017) 7:7. doi: 10.1186/s40405-017-0027-2, PMID: 28944157 PMC5589834

[9] Welte JW Barnes GM Tidwell MCO Wieczorek WF. Predictors of problem gambling in the U.S. J Gambl Stud. (2017) 33:327–42. doi: 10.1007/s10899-016-9639-1 27557549

[10] Izutsu M Suzuki E. Is a sense of community belonging associated with problem gambling in Canada? Soc Psychiatry Psychiatr Epidemiol. (2021) 56:1871–80. doi: 10.1007/s00127-021-02040-w, PMID: 33586005

[11] Williams RJ Shaw CA Belanger YD Christensen DR El-Guebaly N Hodgins DC. Etiology of problem gambling in Canada. Psychol Addict Behav. (2022) 37:483–98. doi: 10.1037/adb0000843 35787101

[12] Okuda M Liu W Cisewski JA Segura L Storr CL Martins SS. Gambling disorder and minority populations: prevalence and risk factors. Curr Addict Rep. (2016) 3:280–92. doi: 10.1007/s40429-016-0108-9, PMID: 28824833 PMC5560497

[13] Raylu N Oei TP. Role of culture in gambling and problem gambling. Clin Psychol Rev. (2004) 23:1087–114. doi: 10.1016/j.cpr.2003.09.005 14729424

[14] Chee TT Lui YS. Pathological gambling, gambling disorder, and problem gambling among the Chinese ethnic population living in Western countries: is culture a sufficient explanation for the reported excess rates? J Gambl Stud. (2021) 37:927–45. doi: 10.1007/s10899-021-10012-7, PMID: 33521910

[15] Mutti-Packer S Hodgins DC Williams RJ Konkolÿ TB. The protective role of religiosity against problem gambling: findings from a five-year prospective study. BMC Psychiatry. (2017) 17:356. doi: 10.1186/s12888-017-1518-5, PMID: 29110644 PMC5674844

[16] Barnes GM Welte JW Tidwell MCO Hoffman JH. Effects of neighborhood disadvantage on problem gambling and alcohol abuse. J Behav Addict. (2013) 2:82–9. doi: 10.1556/JBA.2.2013.004, PMID: 24052815 PMC3775339

[17] Martins SS Storr CL Lee GP Ialongo NS. Environmental influences associated with gambling in Young adulthood. J Urban Health. (2013) 90:130–40. doi: 10.1007/s11524-012-9751-1, PMID: 22895654 PMC3579310

[18] Awaworyi Churchill S Farrell L. Social capital and gambling: evidence from Australia. J Gambl Stud. (2020) 36:1161–81. doi: 10.1007/s10899-019-09901-9, PMID: 31677080

[19] Borkowska M Laurence J. Coming together or coming apart? Changes in social cohesion during the Covid-19 pandemic in England. Eur Soc. (2021) 23:S618–36. doi: 10.1080/14616696.2020.1833067

[20] Lorains FK Cowlishaw S Thomas SA. Prevalence of comorbid disorders in problem and pathological gambling: systematic review and meta-analysis of population surveys. Addiction. (2011) 106:490–8. doi: 10.1111/j.1360-0443.2010.03300.x, PMID: 21210880

[21] Buchanan TW McMullin SD Baxley C Weinstock J. Stress and gambling. Curr Opin Behav Sci. (2020) 31:8–12. doi: 10.1016/j.cobeha.2019.09.004

[22] Hing N Russell AMT Black A Rockloff M Browne M Rawat V. Gambling prevalence and gambling problems amongst land-based-only, online-only and mixed-mode gamblers in Australia: a national study. Comput Hum Behav. (2022) 132:107269–9. doi: 10.1016/j.chb.2022.107269

[23] Bonny-Noach H. Differences between illegal and legal gamblers in Israel: gambling behavior, motivation, and substance use. J. Gambl. Stud. (2022) 39:1239–52. doi: 10.1007/s10899-022-10142-6, PMID: 35780477

[24] Ezrachi Y Harel-Fisch Y Dayan Y Rosiner I. Psychoactive drug use among the adult population in Israel – national epidemiological survey. Jerusalem: Israel Anti-drug Authority (2017).

[25] Mifal Hapais. Responsibe gaming. (2023). Estimating of the risk of addiction to lotteries and gambling. Available at: https://responsiblegaming.pais.co.il/%D7%A1%D7%A7%D7%A8%D7%99%D7%9D-%D7%9E%D7%97%D7%A7%D7%A8%D7%99%D7%9D/%D7%A1%D7%A7%D7%A8-%D7%94%D7%99%D7%A7%D7%A3-%D7%AA%D7%95%D7%A4%D7%A2%D7%AA-%D7%94%D7%94%D7%AA%D7%9E%D7%9B%D7%A8%D7%95%D7%AA-%D7%9C%D7%94%D7%99%D7%9E%D7%95%D7%A8%D7%99%D7%9D-%D7%91%D7%90%D7%A8%D7%A5-%D7%95%D7%91%D7%A2%D7%95%D7%9C%D7%9D/. (Accessed July 12, 2023).

[26] Haklai O. State autonomy marginalization and grievances. In: Brendan O’Leary, editors. Palestinian Ethnonationalism in Israel. Philadelphia, PA: University of Pennsylvania Press (2011). 53–70.

[27] Siegel D. Arab organized crime in Israel. In: Nelen H Siegel D, editors. Organized crime in the 21st century: Motivations, opportunities, and constraints. Cham: Springer International Publishing (2023). 103–19.

[28] Mazbouh-Moussa R Ohtsuka K. Cultural competence in working with the Arab Australian community: a conceptual review and the experience of the Arab council Australia (ACA) gambling help counselling service. Asian J Gambl Issues Public Health. (2017) 7:10. doi: 10.1186/s40405-017-0029-0, PMID: 29250480 PMC5725521

[29] Wardle H Bramley S Norrie C Manthorpe J. What do we know about gambling-related harm affecting migrants and migrant communities? A rapid review. Addict. Behav. (2019) 93:180–93. doi: 10.1016/j.addbeh.2019.01.017, PMID: 30716593

[30] Berrada S Rachidi L El Gnaoui S Agoub M Moussaoui D Battas O. Frequency and risk factors for pathological gambling in a sample of gamblers in Casablanca. Morocco L’encephale. (2009) 35:554–9. doi: 10.1016/j.encep.2008.08.003, PMID: 20004286

[31] Etel C Tabchi S Bou Khalil R Hlais S Richa S. Prevalence of pathological gambling in Lebanese students. Encéphale. (2013) 39:1–5. doi: 10.1016/j.encep.2012.06.015, PMID: 23095599

[32] Guttmann-Steinmetz S Shoshani A Farhan K Aliman M Hirschberger G. Living in the crossfire: effects of exposure to political violence on Palestinian and Israeli mothers and children. Int. J. Behav. (2012) 36:71–8. doi: 10.1177/0165025411406861

[33] Kira I. The development-based taxonomy of stressors and traumas: an initial empirical validation. Psychology. (2021) 12:1575–614. doi: 10.4236/psych.2021.1210099

[34] Steel Z Chey T Silove D Marnane C Bryant RA van Ommeren M. Association of Torture and Other Potentially Traumatic Events with Mental Health Outcomes among Populations Exposed to mass conflict and displacement: a systematic review and Meta-analysis. JAMA. (2009) 302:537–49. doi: 10.1001/jama.2009.1132, PMID: 19654388

[35] Gavriel-Fried B Ajzenstadt M. Securitization vs the yearning for peace in the Israeli casino discourse. Int Gambl Stud. (2013) 13:65–80. doi: 10.1080/14459795.2012.712149

[36] Gavriel-Fried B. Attitudes of Jewish Israeli adults towards gambling. Int Gambl Stud. (2015) 15:196–211. doi: 10.1080/14459795.2015.1012178

[37] Alon S Oren A Blannero K. The first probabilistic web-based panel in Israel. Israeli Sociol. (2022) 23:145–57.

[38] Heiskanen M Toikka A. Clustering Finnish gambler profiles based on the money and time consumed in gambling activities. J Gambl Stud. (2016) 32:363–77. doi: 10.1007/s10899-015-9556-8, PMID: 26026988

[39] Vinberg M Durbeej N Rosendahl I. Gambling and gambling problem among elite athletes and their professional coaches: findings from a Swedish total population survey of participants in four sports. Int Gambl Stud. (2020) 20:262–81. doi: 10.1080/14459795.2020.1726990

[40] Economou M Souliotis K Malliori M Peppou LE Kontoangelos K Lazaratou H. Problem gambling in Greece: prevalence and risk factors during the financial crisis. J Gambl Stud. (2019) 35:1193–210. doi: 10.1007/s10899-019-09843-2, PMID: 31165324

[41] Forsström D Rozental A Sundqvist K. Alcohol use and gambling associated with impulsivity among a Swedish university sample. Int J Environ Res Public Health. (2022) 19:2436. doi: 10.3390/ijerph19042436, PMID: 35206624 PMC8872046

[42] Kroenke K Spitzer RL Williams JBW Löwe B. An ultra-brief screening scale for anxiety and depression: the PHQ–4. Psychosomatics. (2009) 50:613–21. doi: 10.1176/appi.psy.50.6.613 19996233

[43] Israeli Central Bureau of Statistics. Characterization and classification of geographical units by the socio-economic level of the population 2019. (2022). Report no: 375/2022. Available at: https://www.cbs.gov.il/he/mediarelease/DocLib/2022/375/24_22_375b.pdf (Accessed March 13, 2023)

[44] Metcalf O Lawrence-Wood E Baur J Hooff MV Forbes D O’Donnell M. Prevalence of gambling problems, help-seeking, and relationships with trauma in veterans. PLoS One. (2022) 17:e0268346. doi: 10.1371/journal.pone.0268346, PMID: 35613121 PMC9132294

[45] Grubbs JB Kraus SW. Symptoms of problem gambling among US adults who wager on sports. JAMA Netw Open. (2022) 5:e2239670. doi: 10.1001/jamanetworkopen.2022.39670, PMID: 36315149 PMC9623440

[46] Mulkeen J Abdou HA Parke J. A three stage analysis of motivational and behavioural factors in UK internet gambling. Personal Individ Differ. (2017) 107:114–25. doi: 10.1016/j.paid.2016.11.007

[47] Shafir G Peled Y. Citizenship and stratification in an ethnic democracy. In: Moshe Semyonov, editor. Stratification in Israel: Class, ethnicity, and gender. Abingdon, UK: Routledge (2019). 365–84.

[48] Smooha S. Ethnic democracy: Israel as an archetype. Israel Stud. (1997) 2:198–241. doi: 10.2979/ISR.1997.2.2.198

[49] Jamal A. Strategies of minority struggle for equality in ethnic states: Arab politics in Israel. Citizsh Stud. (2007) 11:263–82. doi: 10.1080/17450100701381821

[50] Miller H Thomas S. The “walk of shame”: a qualitative study of the influences of negative stereotyping of problem gambling on gambling attitudes and Behaviours. Int J Ment Heal Addict. (2017) 15:1284–300. doi: 10.1007/s11469-017-9749-8

[51] Pchajek J Edgerton JD Sanscartier M Keough M. Exploring the impact of shame and guilt on coping with gambling problems among emerging adult gamblers. Canad. J. Behav. Sci. (2022) 55:177–88. doi: 10.1037/cbs0000343

[52] Lee GP Ghandour LA Takache AH Martins SS. Investigating the association between strategic and pathological gambling behaviors and substance use in youth: could religious faith play a differential role? Am J Addict. (2014) 23:280–7. doi: 10.1111/j.1521-0391.2014.12101.x, PMID: 24724886

[53] Dickins M Thomas A. Gambling in culturally and linguistically diverse communities in Australia. Melbourne, Australia: Australian Gambling Research Centre (2016).

[54] Rabinowitz D Abu-Baker K. Coffins on our shoulders: the experience of the Palestinian citizens of Israel. Berkeley, CA: Univ of California Press (2005).

[55] Delfabbro P Hundric DD Ricijas N Derevensky JL Gavriel-Fried B. What contributes to public stigma towards problem gambling?: a comparative analysis of university students in Australia, Canada, Croatia and Israel. J. Gambl. Stud. (2022) 38:1127–41. doi: 10.1007/s10899-021-10086-3, PMID: 34800241

[56] Laganà F Elcheroth G Penic S Kleiner B Fasel N. National minorities and their representation in social surveys: which practices make a difference? Qual Quant. (2013) 47:1287–314. doi: 10.1007/s11135-011-9591-1

[57] Lewin-Epstein N. Israel: ISSP 2019 – Social inequality V. Mannheim: International Social Survey Programme (2022).

