# Supplementary material for: Problem gambling severity in a nationally representative sample of the Israeli population: the moderating role of ethnonational affiliation
Source: Front Public Health. 2023 Sep 20;11:1233301. doi: 10.3389/fpubh.2023.1233301 (PMC10548459; doi:10.3389/fpubh.2023.1233301)
Supplement: Supplementary file 1 [file Table_1.docx]

***Supplementary Material***

**Supplementary Tables**

***Table S1: Types of gambling venues in Israel, 2022***

| **Regulator** | **Type** | **Modes** | **Legality** |
| --- | --- | --- | --- |
| Mifal HaPais | Lotto | Land-based gambling venues (domestic) | Legal |
|  | Chance, Pais 777, 123 |  |  |
|  | Scratch cards (Hish Gad, etc.) |  |  |
| Sports Gambling Council | Toto Winner sports betting (basketball games, soccer, tennis, handball, baseball, car races) | Land-based gambling venues (domestic)  Online | Legal |
| Unregulated providers | Sports bets other than through Toto Winner | Land-based gambling venues (domestic or international)  Online | Legal (for individuals gambling in international land-based venues)  Illegal (for individuals gambling in domestic land-based venues and online) |
|  | Horse racing |  |  |
|  | Slot machines |  |  |
|  | Table games (roulette, craps, etc.) |  |  |
|  | Card games (poker, blackjack, etc.) |  |  |
|  | Bingo for money |  |  |
|  | Gambling on the stock market | Land-based gambling venues (domestic or international)  Online | Legal |

***Table S2: Risk factors for PGS moderated by ethnonational affiliation (Arab) among Israelis who engaged in gambling, 2022 – full results***

| **Reference category: Non-problem gamblers** | **Low-risk gamblers**  **Odds ratio (95% CI)** | **Problem and moderate-risk gamblers**  **Odds ratio (95% CI)** |
| --- | --- | --- |
| Constant | 0.20 (0.13-0.32) | 0.07 (0.04-0.13) |
| Locality's socioeconomic cluster × Arab | 0.55 (0.26-1.17) | 0.65 (0.28-1.54) |
| Neighborhood cohesion scale × Arab | 5.04 (2.51-10.14) | 2.53 (1.06-6.05) |
| Depression × Arab | 1.67 (0.32-8.81) | 0.02 (0.00-0.17) |
| Anxiety × Arab | 0.24 (0.05-1.18) | 1.62 (0.27-9.56) |
| Self-reported stress × Arab | 0.55 (0.30-1.02) | 0.72 (0.36-1.43) |
| Illegal online gambling × Arab | 16.82 (0.51-554.13) | 231.01 (7.14-7469.17) |
| Gambling behaviors × Arab | 4.45 (1.93-10.29) | 2.47 (0.93-6.58) |
| **Sociodemographic variables** |  |  |
| Gender (man vs. woman) | 1.06 (0.78-1.44) | 1.48 (0.98-2.24) |
| Ethnonational affiliation (Israeli Arab/Jewish) | 2.86 (1.00-8.14) | 5.75 (1.77-18.71) |
| *Self-perceived religiosity (reference category: secular)* |  |  |
| Traditional | 1.23 (0.89-1.71) | 1.79 (1.16-2.77) |
| Religious or very religious | 1.00 (0.64-1.57) | 1.19 (0.64-2.21) |
| Education (academic vs. non-academic) | 0.79 (0.57-1.08) | 0.70 (0.45-1.08) |
| *Labor market status (reference category: employed)* |  |  |
| Unemployed | 0.71 (0.31-1.62) | 2.29 (1.04-5.02) |
| Not in the labor force | 1.30 (0.89-1.91) | 0.81 (0.47-1.39) |
| *Age group (reference category: 18-29)* |  |  |
| 30-39 | 1.15 (0.74-1.79) | 0.93 (0.54-1.61) |
| 40-64 | 0.85 (0.57-1.25) | 0.65 (0.40-1.06) |
| 65+ | 1.48 (0.87-2.52) | 1.55 (0.77-3.13) |
| **Environmental variables** |  |  |
| Locality's socioeconomic cluster | 0.91 (0.77-1.09) | 0.96 (0.76-1.22) |
| Neighborhood cohesion scale | 0.92 (0.78-1.07) | 0.89 (0.72-1.10) |
| **Psychopathological variables** |  |  |
| Depression | 1.22 (0.85-1.76) | 1.39 (0.86-2.25) |
| Anxiety | 1.23 (0.79-1.92) | 1.22 (0.69-2.19) |
| Self-reported stress level | 1.26 (1.05-1.52) | 1.30 (1.01-1.67) |
| **Gambling-related variables** |  |  |
| Legal online gambling | 1.77 (1.16-2.69) | 2.38 (1.44-3.94) |
| Illegal online gambling | 1.32 (0.71-2.43) | 3.94 (2.15-7.22) |
| Gambling behavior | 1.77 (1.50-2.09) | 1.91 (1.58-2.31) |

*Notes: In this multinomial logit regression, "non-problem gamblers" was the reference category. The data were weighted by level of education and ethnonational affiliation (Israeli Jewish/Arab).*
